# Quarterly Report (No. 3,) of the Medical and Surgical Practice of the St. George's and St. James's General Dispensary, 14, Old Burlington-Street; from January 13, to April 12, 1823, Inclusive

**Published:** 1823-05

**Authors:** George Gregory

**Affiliations:** Senior Physician to the Dispensary, &c. &c.


### STATISTICAL MEDICINE.

ART. I.—*Quarterly Report (No.* 3,*) of the Medical and Surgical Practice of the St. George's and St. James's General Dispensary,* 14, *Old Burlington-street; from January* 13, *to April* 12, 1823, *inclusive.* By GEORGE GREGORY, M.D. Senior Physician to the Dispensary, &c. &c.

| | | | |
|---|---|---|---|
| Acute Constitutional Affections | Fever, Common Continued | 44 | |
| | —— Intermittent | 7 | |
| | —— Remittent, of Children | 15 | |
| | —— Bilious | 8 | |
| | Typhus | 1 | |
| | Variola | 3 | |
| | Measles | 1 | |
| | Rheumatism, Acute and Subacute | 17 | 96 |
| Acute and Chronic Diseases of the Head and Nervous System | Apoplexy (threatened) | 2 | |
| | Epilepsy | 3 | |
| | Phrenitis and Hydrocephalus | 7 | |
| | Head-ache and Giddiness | 14 | |
| | Fatuitas | 1 | |
| | Neuralgia Tibiæ | 1 | |
| | Ophthalmia | 5 | |
| | Amaurosis | 1 | |
| | Tremor | 1 | 35 |
| Acute and Chronic Diseases of the Thorax | Pneumonia | 18 | |
| | Catarrh and Cynanche | 20 | |
| | Bronchitis, Subacute and Chronic | 64 | |
| | Phthisis Pulmonalis | 11 | |
| | Hydrothorax | 6 | |
| | Angina Pectoris | 1 | |
| | Aneurisma Aortæ | 1 | |
| | Pertussis | 7 | |
| | Pain of the Side | 5 | |
| | Asthma Dyspepticum | 4 | 137 |
| Diseases of the Chylopoietic Viscera | Enteritis | 1 | |
| | Dyspepsia | 62 | |
| | Hepatitis | 4 | |
| | Jaundice | 3 | |
| | Incessant Vomiting | 1 | |
| | Hæmatemesis | 1 | |
| | Hæmorrhoids | 1 | |
| | Constipation | 1 | |
| | Worms | 1 | |
| | Diarrhœa | 12 | 87 |
| Diseases of the Urinary System | Nephralgia | 2 | |
| | Incontinence of Urine | 2 | |
| | Diseases of the Testicle | 9 | |
| | Diseased Prostate | 1 | |
| | Stricture | 2 | 16 |

| | | | |
|---|---|---|---|
| Diseases of the Uterine System | Irritation from Pregnancy | 1 | |
| | Menorrhagia | 2 | |
| | Leucorrhœa | 1 | |
| | Amenorrhœa | 11 | |
| | Dysmenorrhœa | 2 | |
| | Hysteria | 1 | |
| | Carcinoma Uteri | 1 | |
| | Diseased Vagina | 1 | |
| | Enlarged Nympha | 1 | = 21 |
| Chronic Constitutional Affections | Scrofula | 10 | |
| | Rickets | 1 | |
| | Plethora | 3 | |
| | Cachexia | 3 | |
| | Hypochondriasis | 2 | |
| | Debility | 2 | |
| | Dropsy | 4 | = 25 |
| Chronic Cutaneous Diseases | Lichen | 2 | |
| | Reue | 1 | |
| | Herpes | 2 | |
| | Lepra and Psoriasis | 4 | |
| | Porrigo | 3 | |
| | Erythema | 1 | |
| | Impetigo | 1 | |
| | Lupus | 1 | |
| | Psora | 2 | |
| | Eruptions not well characterized | 5 | = 22 |
| Syphilitic Affections | Syphilis | 3 | |
| | Pseudo-Syphilis | 1 | |
| | Gonorrhœa | 5 | |
| | Cachetic Sore Throat | 2 | |
| | Syphilitic Lichen | 1 | |
| | Bubo and Chancre | 7 | = 19 |
| Diseases strictly Local | Fractures and Dislocations | 4 | |
| | Bruises, Sprains, and slight Injuries | 24 | |
| | Diseases of the Mamma | 6 | |
| | Phlegmons, Abscesses, and Ulcers | 41 | |
| | Affections of the Joints | 7 | |
| | Herniæ | 2 | |
| | Diseases of the Nose and Face | 5 | |
| | Fistula | 1 | |
| | Deafness | 3 | |
| | Caries of Bones | 2 | = 95 |
| | Cases not reported | | 9 |
| Total | Under the care of the Physicians | 352 | 561 |
| | ——— Surgeons | 160 | |
| | ——— Surgeon-Accoucheur | 49 | |

The admissions during the last quarter have been more than commonly numerous; and, from the preceding table, it will be seen that disease has presented itself under a very great variety of aspects. Catarrhal and bronchial complaints, attended with unusually severe symptoms, have been prevalent in all parts of the town; and these, therefore, constitute a very large proportion of the admissions during the last quarter. The disorder possessed many of the characters of genuine influenza. It was distinguished by its sudden accession, the great debility and languor which attended it, and the tedious convalescence by which it was followed. In many cases the mucous membrane of the bowels was implicated, and diarrhœa became a leading and very troublesome symptom. In a few instances, the catarrhal affection merged in actual pneumonia, requiring a free use of the lancet. It has been intimated to me, that a complaint of the same character has prevailed extensively among horses, and that there has been a great mortality among those animals, particularly along the banks of the Thames.

It is a singular circumstance, that, while diseases traceable in almost all instances to the variable state of the weather have been so common, others of a *specific* character have been proportionably rare. Only nineteen patients having small-pox have been admitted into the Small-Pox Hospital between the 20th of February and the 12th of April, and of them two only have died; whereas, from the 1st of January to the 19th of February, thirty-three were admitted, of whom fourteen died. On comparing these numbers with the admissions during the year 1803, when the *influenza* prevailed, it appears that the same phenomenon was then observable. Between the 1st of March and the 31st of August of that year, seventeen patients only were admitted, of whom but three died.

Several cases of genuine ague, originating in this town, have come under treatment during the last quarter. Jaundice also, and bilious complaints generally, have been more frequent than is usual at this season of the year. The mortality has been great in all parts of the town. It is generally stated, that a more sickly season has never been known. Seventeen deaths have occurred in the practice of the Dispensary, which may be thus arranged:—Bronchitis Senilis, 4; Phthisis Pulmonalis, 3; Hydrothorax, 4; Infantile Fever, 1; Infantile Pneumonia, 1; Hæmatemesis, 1; Encephalic Disease, 1; Measles, 1; Diseased Prostate, 1.

In one of the cases of hydrothorax, a remarkable appearance presented itself, which may be worth recording. The patient had been labouring under thoracic symptoms for nearly three years. On dissection, the pericardium was found so enormously distended, that, when the sternum was removed, no portion of the lungs was discernible. It contained eighteen, or perhaps twenty, ounces of serum; besides a heart very greatly enlarged.

